# Impairment of the visuospatial working memory in the patients with Parkinson’s Disease: an fMRI study

**DOI:** 10.1186/s12883-021-02366-7

**Published:** 2021-09-03

**Authors:** Shoji Kawashima, Yoko Shimizu, Yoshino Ueki, Noriyuki Matsukawa

**Affiliations:** 1 Department of Neurology and Neuroscience, Graduate School of Medical Science, Nagoya City University, 1 Kawasumi, Mizuho-ku, 467-8601 Nagoya, Japan; 2 Department of Rehabilitation Medicine, Graduate School of Medical Science, Nagoya City University, 1 Kawasumi, Mizuho-ku, 467-8601 Nagoya, Japan

**Keywords:** Mild cognitive impairment, Parkinson’s disease, functional MRI, working memory, n-back test

## Abstract

**Background:**

Mild cognitive impairment (MCI) is a common symptom in the patients with Parkinson’s disease (PD). The characteristics of cognitive impairment in PD are executive function (including working memory) and visuo-perceptual processing. The visuospatial n-back test has the merit of minimizing the influence of educational biases involved in the verbal n-back test. Furthermore, it can assess both visuospatial recognition and working memory in a single test.

**Methods:**

We aimed to clarify the advantage of the visuospatial n-back test as a tool for detecting impairments of working memory in PD. We enrolled 28 right-handed patients with PD (18 males, 10 females) and 12 age-matched healthy controls (HC; 7 males, 5 females). Thirteen patients were classified as MCI (PD-MCI), and 15 as cognitively normal PD (PD-CN). Using functional MRI (fMRI), we explored the specific brain regions associated with the performance of the n-back test in the PD-MCI, PD-CN, and HC groups. The 0-back test assesses visuospatial recognition, while the 1-back and 2-back tests assess visuospatial working memory. Group comparisons were performed for three loads of this test.

**Results:**

Patients with PD performed significantly worse in terms of the correct answer rates of all n-back tests compared with HC. fMRI analyses performed during the 2-back test revealed reduced activation in the bilateral dorsolateral prefrontal cortex, middle frontal gyrus (MFG), and parietal lobule in the PD group compared with the HC group. In contrast, the fMRI result during the 0-back test showed only a marginal difference in the frontal lobe. On comparisons of task performance between the PD-MCI and PD-CN groups, we found that the correct answer rate in the 2-back test was lower in the PD-MCI group than in the PD-CN group. However, scores of the 0-back and 1-back tests were not significantly different between the two groups. The fMRI findings revealed that activations within the middle frontal gyrus (MFG) and inferior parietal lobule (IPL) during the 2-back test were reduced in the patients with PD-MCI when compared to those with PD-CN.

**Conclusions:**

This study reports reduced activation of the MFG and IPL in patients with PD-MCI. These regions may be associated with the pathophysiology of working memory impairment in patients with PD, which involves fronto-striatal network dysfunction.

## Introduction

Striatal dopamine depletion due to the degeneration of nigrostriatal dopaminergic neurons causes motor disturbances in the patients with Parkinson’s disease (PD). The cognitive impairment is common symptoms of PD as well as motor disturbances. The movement disorder society (MDS) has published the clinical criteria for dementia in PD (PDD) in 2007 [1], and the MDS Task Force has provided the criteria for mild cognitive impairment (MCI) in PD (PD-MCI) in 2012 [2]. MCI is a transitional stage between normal ageing and dementia that has been used to detect and treat early dementia [3]. In a recent cohort study, 20 % of *de novo* patients with PD were classified as PD-MCI at baseline, and 39 % of patients with baseline or incident PD-MCI progressed to dementia during the 5-year follow-up period [4]. The MDS Task Force has advocated two criteria: Level I (abbreviated) criteria based on the Montreal Cognitive Assessment (MoCA) score for global cognitive function, and Level II (comprehensive) criteria based on an efficient neuropsychological test battery of subdomains. Although the different criteria produce different classifications of PD-MCI status, both assessment (based on abbreviated and comprehensive criteria), are needed to evaluate cognitive impairment of the PD.

With regards to cognitive impairment in PD, various studies have reported impairments in frontal executive function (including working memory) [5], and visuo-perceptual processing [6]. Working memory is responsible for the short-term storage and online manipulation of information necessary for higher cognitive function [7]. Impaired working memory can disrupt activities of daily living. One major test to assess working memory is the n-back test, which was developed in the 1950 s by Kirchner. Briefly, subjects are presented a sequence of stimuli one-by-one, and they must decide and react immediately if the currently presented stimulus is identical to that presented N trials ago [8].

Many functional MRI (fMRI) studies have used the verbal n-back task to explore brain activation associated with working memory processing. A recent quantitative meta-analysis of 96 primary studies that used the n-back task showed that performance in the n-back task is associated with the activation of the bilateral frontal and cortical regions (i.e. middle frontal gyrus, inferior parietal lobule, precuneus, left superior frontal gyrus, left anterior insula) [9]. Further, task performance in working memory is associated with dopaminergic neurotransmission in the striatum [10, 11]. Focusing on neuroimaging studies of cognitive impairment in PD using the 2-back test, Ekman et al. reported decreased test performance in patients diagnosed with PD-MCI when compared with PD patients without MCI; this decrease was associated with reduced levels of dopamine transporter binding in the right caudate nucleus, as measured by single photon emission tomography [12]. More specifically, they revealed that isotope uptake in the right caudate correlates with striatal fMRI blood-oxygen-level-dependent signals in patients with PD-MCI. Similarly, Lewis et al. reported that the verbal working memory in early-stage PD was accompanied by reduced activity in the fronto-striatal neural circuitry [13]. However, in terms of the cortical regions reported in the literature, patients with PD have been reported to present different neuroimaging findings associated with impaired verbal working memory. Such diversity may be derived from differences in patient sample characteristics, such as education level or disease duration.

Focusing on visuo-perceptual dysfunction, Uc et al. reported that patients with PD showed impaired visual perception, compared with elderly controls, and that visual dysfunction contributes to parkinsonian disability through its influences on cognition and locomotion [14]. Furthermore, Bradley et al. reported that patients with PD were significantly slower than the control group when performing a visuospatial working memory task; however, these patients were not significantly slower and did not make more errors in the verbal task [15]. To assess the influence of aging on visuospatial working memory in normal subjects, several fMRI studies have used the visuospatial version of the n-back task as well as the verbal n-back task [16, 17]. Considering these studies, we focused on this test because it has two advantages for the cognitive assessment of patients with PD: first, the merit of minimising the influence of educational biases or differences in native language, such as those found in the verbal n-back test; and second, it simultaneously assesses visuospatial recognition in a single test.

Therefore, the purpose of this study was to use fMRI to explore the differences in brain regions associated with the performance of the visuospatial version of the n-back test in patients with PD-MCI and cognitively normal PD (PD-CN), using voxel-based analysis. We also aimed to assess the relationship between task-related cortical regions and the performance of visuospatial n-back tests in patients with PD-MCI.

## Methods

### Participants

All subjects were recruited from the Department of Neurology at Nagoya City University Hospital. We enrolled 28 right-handed patients with PD (18 males, 10 females) and 12 age-matched healthy controls (HC; 7 males, 5 females) in the study. We excluded participants if they had depression, severe insomnia, severe hearing loss, or any other disease that might severely interfere with the fMRI.

All patients with PD fulfilled the United Kingdom Parkinson’s Disease Brain Bank Criteria for clinical diagnosis [18]. Patients were excluded if they had dementia according to the criteria for PD dementia provided by the Movement Disorder Society Task Force [1]. No patients had a disease other than PD that affected motor and cognitive function. Motor symptom was assessed with motor section of Unified Parkinson’s Disease Rating Scale (UPDRS part3) [19]. The study was approved by the local Ethical Committee and complied with national legislation and the Declaration of Helsinki guidelines. All participants provided written informed consent prior to the data acquisition.

### Neuropsychological test and classification of PD-MCI

Movement disorder specialists performed the neuropsychological test battery in all participants. Global cognitive function was assessed with MMSE and MoCA. Psychomotor speed and attention were tested with Trail-Making Test Part A (TMT-A) and the Paced Auditory Serial Addition Test (PASAT). Executive function and rapid set shifting were tested with Trail-Making Test Part B (TMT-B) and Clock Drawing Test. For scoring Clock Drawing Test, we used the Sunderland scoring system which consisting of a single 10-point rating, with higher numbers indicating better performance [20]. Memory was tested using the 3 trials of word list leaning and delayed recall of the Auditory Verbal Learning Test (AVLT). Language was tested with the Verbal Fluency Test of animal naming and phonological recall (total number of words in 60 s). Visuospatial function was tested with the visuospatial version of 0-back test and intersecting pentagons from the MMSE.

Using these tests, PD-MCI was classified according to the Level II criteria of the Movement Disorder Society Task Force which advocated the detection thresholds of -2 SD had significant impact on the discriminative validity of all measures [21]. In accordance with this criteria, PD-MCI was defined when patients’ scores were 2 SD below the normative mean score of the neuropsychological assessments, and it was defined when their impairment on at least two tests represented by either two impaired tests in one cognitive domain (single domain impairment) or one impaired test in two different cognitive domains (multi domain impairment) [2]. The patients were classified as PD-CN when scores were within 2 SD of the normative mean score.

### Visuospatial n-back test

The test used in this study is a modified version of visuospatial n-back test which was reported in fMRI studies for normal subjects [16, 17]. The subjects were asked to perform the tests with 3 load levels during the fMRI. The stimuli were white squares randomly presented in 1 of 8 spatial locations on a screen, through a mirror positioned on a head-coil. The presentation of the stimuli was controlled by a program (Presentation software) that initiated the acquisition of the MRI and the behavioural data. They were instructed to pay attention to a sequence of visual stimuli and press a pre-defined button, as fast as possible. For the 0-back test, the subjects were instructed to press the left button with their index finger when a white square was presented in the left-upper and center-upper position. They were instructed to press the right button when the stimulus appeared in any other location. For the 1-back test, they were instructed to press the left button whenever a stimulus was presented in the same location as the previous stimulus. For the 2-back test, they had to press the left button whenever a stimulus was presented in the same location as the two trials previous. When the stimulus was presented in any other location during the 1-back and 2-back test, the patients were instructed to press the right button with the middle finger. The higher the number n requires the higher level of attention and working-memory.

### Imaging protocol

**Fig. 1:**
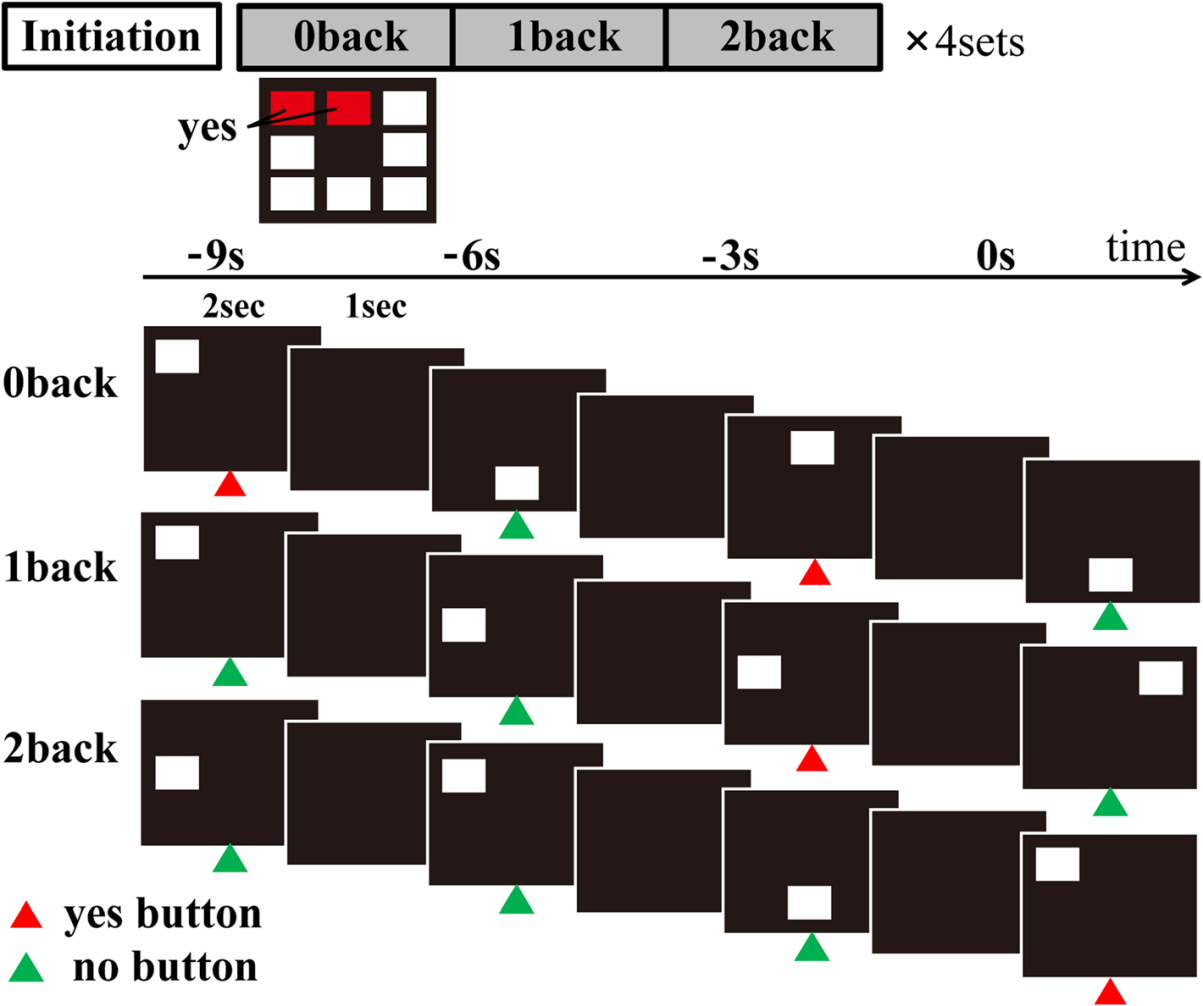
Experimental protocol. The figure shows the protocol of n-back task used in this study. To detect the activated brain regions associated with the visuospatial n-back task, we used a blocked design fMRI, alternating n-back task conditions and rest conditions. In n-back task conditions, the white square was presented for 2 s at random in 1 of 8 possible locations on screen (exposure time), and black screen was presented for 1 s after the presentation of stimuli (interstimuli interval). Therefore, the consecutive stimuli were presented every 3 s. In the 0-back test, the subjects were instructed to press the left button with their index finger when the white square was presented in predetermined locations. They were instructed to press the right button when the stimulus appeared in any other location. In the 1-back test, the subjects were instructed to press the left button when the stimulus presented in the same location as the previous one. In the 2-back test, they were instructed to press the left button whenever a stimulus was presented in the same location as the two trials previous. When the stimulus was presented in any other location during the 1-back and 2-back test, the patients were instructed to press the right button with the middle finger

To detect brain regions activated in the subjects performing the visuospatial n-back test, we used the block-design fMRI, which alternated between n-back test and rest conditions. For n-back test conditions, the white square was randomly presented for 2 s in 1 of 8 possible locations on screen; a black screen was presented for 1 s after stimulus presentation. Each test condition consisted of 15 trials over the course of 45 s; each rest condition lasted for 15 s. During a single scan, each test condition was repeated 4 times, in numerical order (0-1-2 back). Thus, each condition included 60 trials (Fig. 1). Visuospatial n-back test was performed during the ‘On’ state to avoid cognitive change or anxiety arising from being in the ‘Off’ state during testing.

### Image data acquisition and analysis

All MRI were acquired with a Siemens Skyra syngo MR E11 3.0 T scanner (Siemens, Germany). High-resolution T1-weighted images were acquired via volumetric 3D spoiled gradient recall sequence. Acquisition parameters were as follows: repetition time (TR) = 1900 ms, echo time (TE) = 2.43 ms, flip angle (FA) = 9, field of view (FOV) = 256 × 256 mm, slice thickness = 1 mm, slice gap = 0, voxel size = 1 × 1 × 1 mm, number of slices = 176. The fMRI measurements were performed using a gradient echo EPI sequence: repetition time (TR) = 2500 ms, echo time (TE) = 30 ms, slice thickness = 3 mm, total 149 volumes, with matrix size of 64 × 64 and field of view of 192 × 192 mm, resulting in voxel size of 3 × 3 × 3 mm.

All the images were pre-processed and analysed with Matlab (version 8.1, The Mathworks Inc, Natick, MA) and SPM8 software (Department of Cognitive Neurology, London). Images were realigned to correct for movement. Every axis of scans was checked for possible movement. None of the participants demonstrated scan-to-scan head movements > 4mm. Scans were co-registered and realigned to the first volume to correct head translation or rotation during the scanning. The realigned images were normalised to Montreal Neurologic Institute (MNI) space. The transformed image data were smoothed with a Gaussian philtre (full width at half-maximum = 10 mm).

The image data were analysed with a random effect procedure and a parametric model to identify the brain areas where the activation correlated with the task. We specified the first level analysis model, estimated and defined the parameters and t-contrasts for n-back test conditions vs. the rest condition. The resulting contrast maps from each contrast and for each subject were then used in a second level random effects analysis for between groups effects (PD vs. HC, and PD-MCI vs. PD-CN). Group comparisons were performed for 3 loads of the n-back test, the 1-back vs. 0-back condition and the 2-back vs. 0-back condition. Further, the correlations between score of the n-back test and task-related activation were analysed for all patients with PD-MCI. The statistical significance was established at *P* < 0.01 (uncorrected) with cluster size > 50 voxels in group analysis, and *P* < 0.001 (uncorrected) with cluster size > 50 in correlation analysis.

### Statistical analysis

To examine group differences in the clinical profiles between PD-MCI and PD-CN, each profile (age, UPDRS part3, dosage of L-DOPA, L-dopa equivalent daily dose of dopamine agonist (LEDD) [22], MMSE, MoCA, time to complete TMT, PASAT, AVLT, verbal fluency test), were compared using the independent t-tests as appropriate. The group difference in the Hoehn and Yahr Scale was analysed using the Mann-Whitney U test. The scores on n-back tests and reaction times were analysed using one-way analysis of variance (ANOVA), because each load level required several neuropsychological cognitive domains. In the PD-MCI group, correlation between scores on the n-back test and other factors were analysed using Pearson’s correlation analysis. SPSS version 15.0 was used for these statistical analyses. *P* < 0.05 was considered significant.

## Results

### Clinical profiles of the patients with PD and HC

The demographic and neuropsychological profiles of the patients with PD and HC are summarised in Table 1. On comparisons of global cognition, the MMSE and MoCA scores were both significantly lower in PD patients than in HC (MMSE: *P* < 0.05, MoCA: *P* < 0.001). On comparisons of neuropsychological subdomains, scores of the PASAT, delayed recall of AVLT, word list learning of AVLT, Verbal Fluency Test, and Clock Drawing Test were all lower in PD patients comparing with HC (PASAT: *P* < 0.05, delayed recall of AVLT: *P* < 0.01, word list learning of AVLT: *P* < 0.01, Verbal Fluency Test: *P* < 0.05, Clock Drawing Test: *P* < 0.05). Patients with PD took a significantly longer time to complete the TMT-B than HC (*P* < 0.05). Furthermore, the patients with PD had significantly inferior performance of the correct answer rates of the n-back tests comparing with HC; the correct answer rate in the 0-back test was 85.4 ± 16.9 % in patients with PD and 97.5 ± 4.9 % in HC (*P* < 0.05); that in the 1-back test was 66.9 ± 22.1 % in patients with PD and 87.0 ± 13.2 % in HCs (*P* < 0.05); and that in the 2-back test was 56.5 ± 21.9 % in patients with PD and 73.5 ± 16.7 % in HC (*P* < 0.05).

**Table 1:** The demographic and neuropsychological profiles of the patients with PD and HC

	PD	HC	
	***n* = 28**	***n* = 12**	***P*value**
Age	68.8 ± 4.2	68.3 ± 2.3	N.S.
Men	18	6	N.S.
MMSE	27.4 ± 2.2	29.8 ± 0.3	*P* < 0.05
MoCA	22.8 ± 3.6	28.8 ± 1.8	*P* < 0.001
TMT-A (second)	56.9 ± 25.9	37.8 ± 18.7	N.S.
TMT-B (second)	193.6 ± 120.0	99.1 ± 26.6	*P* < 0.05
PASAT	24.0 ± 7.1	36.5 ± 10.3	*P* < 0.05
AVLT (delayed recall)	7.3 ± 3.5	10.9 ± 2.2	*P* < 0.01
AVLT (word list learning)	5.8 ± 2.8	8.9 ± 1.7	*P* < 0.01
Verbal fluency (words)	13.9 ± 3.0	16.2 ± 4.1	*P* < 0.01
Clock Drawing Test	9.1 ± 1.1	9.9 ± 0.3	*P* < 0.05
0-back test (%)	85.4 ± 16.9	97.5 ± 4.9	*P* < 0.05
1-back test (%)	66.9 ± 22.1	87.0 ± 13.2	*P* < 0.05
2-back test (%)	56.5 ± 21.9	73.5 ± 16.2	*P* < 0.05

*MoCA* Montreal Cognitive Assessment, *PASAT* Paced Auditory Serial Addition Test; *AVLT* Auditory Visual Learning Test; *N.S.* not significant

### Clinical profiles of the patients with PD-MCI and PD-CN

The clinical profiles of patients with PD-MCI and PD-CN are summarised in Table 2. A total of 13 patients were classified as PD-MCI); 15 patients were classified as PD-CN. The UPDRS motor scores of the patients with PD-MCI were significantly higher than those of the patients with PD-CN (*P* < 0.001). Age, Hoehn and Yahr stage, and disease duration did not differ between groups (Age: *P* = 0.177, Hoehn and Yahr stage: *P* = 0.844, disease duration: *P* = 0.758). There were no differences among the groups in daily doses of L-DOPA (*P* = 0.421), total L-DOPA and L-DOPA equivalent daily dose of dopamine agonist (LEDD) (*P* = 0.895). In the comparisons of global cognition, there were no group difference in MMSE scores (*P* = 0.096); however, the MoCA scores were significantly lower in the patients with PD-MCI in those with PD-CN (*P* < 0.01). On comparisons of neuropsychological subdomains, scores of the PASAT, delayed recall of AVLT, word list learning of AVLT, and Verbal Fluency Test were lower in the patients with PD-MCI in those with PD-CN (PASAT: *P* < 0.05, delayed recall of AVLT: *P* < 0.01, word list learning of AVLT: *P* < 0.01, Verbal Fluency Test: *P* < 0.01). Patients with PD-MCI took a significantly longer time to complete the TMT-A and TMT-B (TMT-A: *P* < 0.01, TMT-B: *P* < 0.001). There was no statistical difference in the score of Clock Drawing Test between groups (*P* = 0.17). Furthermore, the patients with PD-MCI had poorer performance in terms of the correct answer rates of the n-back tests comparing with PD-CN; the correct answer rate in the 0-back test was 79.2 ± 18.5 % in PD-MCI and 89.3 ± 15.2 % in PD-CN (*P* = 0.124); that in the 1-back test was 56.5 ± 20.4 % in PD-MCI and 71.7 ± 24.9 % in PD-CN (*P* = 0.094); and that in the 2-back test was 45.8 ± 20.4 % in PD-MCI and 63.7 ± 18.3 % in PD-CN (*P* < 0.05).

**Table 2:** Baseline characteristics of the patients with PD-MCI and PD-CN

	PD-MCI	PD-CN	
	***n* = 13**	***n* = 15**	***P*value**
Age	69.4 ± 2.8	67.3 ± 4.6	N.S.
Men	8	10	N.S.
Disease Duration (year)	5.0 ± 3.2	5.4 ± 3.5	N.S.
HY stage	2.1 ± 0.8	2.1 ± 0.7	N.S.
UPDRS part3	19.8 ± 8.6	10.1 ± 5.2	*P* < 0.001
L-DOPA (mg)	262 ± 153	223 ± 90	N.S.
LEDD (mg)	296 ± 199	287 ± 151	N.S.
MMSE	26.9 ± 1.8	28.1 ± 1.9	N.S.
MoCA	21.1 ± 3.0	24.3 ± 2.8	*P* < 0.01
TMT-A (second)	65.6 ± 21.0	43.8 ± 11.2	*P* < 0.01
TMT-B (second)	276 ± 107.5	111 ± 50.4	*P* < 0.001
PASAT	21.2 ± 6.1	27.2 ± 6.2	*P* < 0.05
AVLT (delayed recall)	5.5 ± 3.2	9.1 ± 2.9	*P* < 0.01
AVLT (word list learning)	4.2 ± 2.5	7.5 ± 2.2	*P* < 0.01
Verbal fluency (words)	12.0 ± 2.9	15.5 ± 3.1	*P* < 0.01
Clock Drawing Test	8.8 ± 1.3	9.3 ± 0.8	N.S.
0-back test (%)	79.2 ± 18.5	89.3 ± 15.2	N.S.
1-back test (%)	56.5 ± 20.4	71.7 ± 24.9	N.S.
2-back test (%)	45.8 ± 20.4	63.7 ± 18.3	*P* < 0.05

*HY stage* Hoehn and Yahr stage; *UPDRS part3* motor sections of United Parkinson’s Disease Rating Scale; *LEDD* L-dopa equivalent daily dose of dopamine agonist. Calculation of LEDD for each patient was based on theoretical equivalence to L-dopa as follows: L-dopa dose + L-dopa dose × 1/3 [if on entacapone + bromocriptine (mg) ×10 + cabergoline or pramipexole (mg) ×67 + ropinirole (mg) ×20 + pergolide (mg) ×100 + apomorphine (mg) ×8]. MoCA, Montreal Cognitive Assessment, PASAT, Paced Auditory Serial Addition Test; AVLT, Auditory Visual Learning Test; N.S., not significant

### Visuospatial n-back test in the patients with PD

**Fig. 2:**
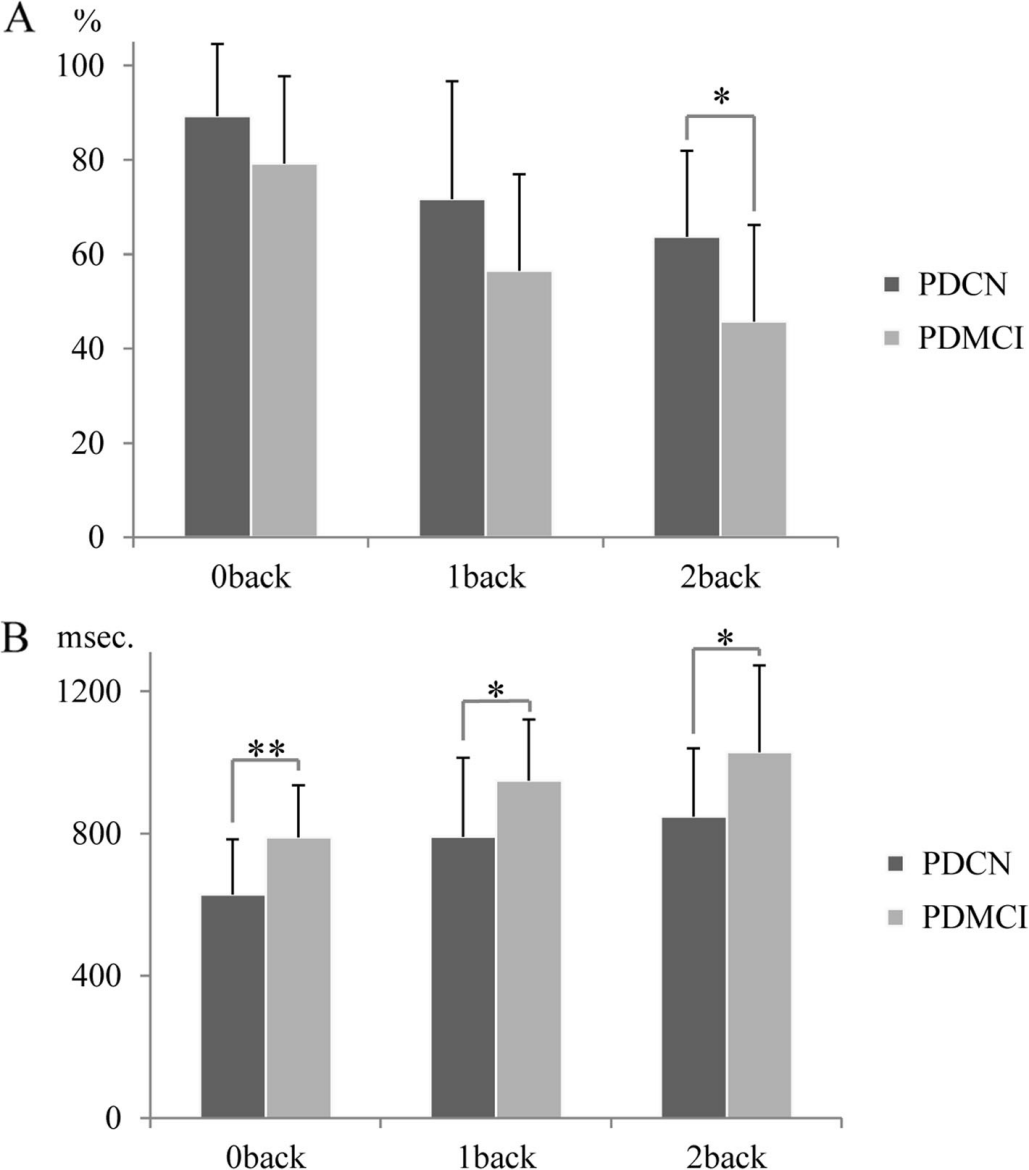
Performance of the visuospatial n-back test in the patients with PD. **A**. The figure shows rates of correct answers for the patients with PD-CN and PD-MCI. The scores on the 0-back test did not differ significantly between groups. The scores on the 2-back test of PD-MCI were significantly lower than that of PD-CN. **B**. The figure shows the response time to press correct button. The patients with PD-MCI took significantly longer time to respond in all task conditions. * *P* < 0.05, ** *P* < 0.01

The reaction times and correct answer rates for patients with PD are summarised in Fig. 2. The reaction times of all n-back tests were significantly longer in the patients with PD-MCI, compared with the PD-CN (0-back: F(1, 26) = 7.873, *P* < 0.01, 1-back: F(1, 26) = 4.348, *P* < 0.05, 2-back: F(1, 26) = 4.792, *P* < 0.05). In the correct answer rate, there was a significant decrease of the score of 2-back test in the patients with PD-MCI, compared with the PD-CN (F(1, 26) = 6.001, *P* < 0.05). Scores of the 0-back and 1-back tests were not different between the groups (0-back: F(1, 26) = 2.519, *P* = 0.121; 1-back: F(1, 26) = 3.013, *P* = 0.094).

### Correlation between scores of the n-back test and clinical profiles of the patients with PD-MCI

The correlations between scores of the n-back test and other factors of the patients with PD-MCI are presented in Table 3. There was a significant negative correlation between the scores of the 1-back test and the score of UPDRS Part 3 (*R*=-0.41, *P* < 0.05) and between the scores of the 2-back test and UPDRS Part 3 (*R*=-0.42, *P* < 0.05). Additionally, we found positive correlations between the scores of MoCA and the 1-back test (*R* = 0.39, *P* < 0.05), as well as between the scores of MoCA and the 2-back test (*R* = 0.41, *P* < 0.05). We also found a negative correlation between the time to complete TMT-B and the scores of 0-back test (*R*=-0.50, *P* < 0.01), however, there was no correlation between the time to complete TMT-A and any load of the n-back test.

**Table 3:** Correlations between scores of the n-back test and clinical profiles of the PD-MCI group

		0-back	1-back	2-back	PASAT	TMT-B
**MoCA**	Pearson’ R		0.39	0.413	0.35	−0.54
	*p* value	N.S.	0.040	0.029	0.041	0.003
**TMT-A**	Pearson’ R					0.524
	*p* value	N.S.	N.S.	N.S.	N.S.	0.004
**TMT-B**	Pearson’ R	−0.495	−0.5		−0.5	1
	*p* value	0.007	0.007	N.S.	0.007	
**UPDRS part3**	Pearson’ R		-0.406	-0.415	−0.406	0.685
	*p* value	N.S.	0.032	0.028	0.032	0.001

*N.S.* not significant

### fMRI

**Fig. 3:**
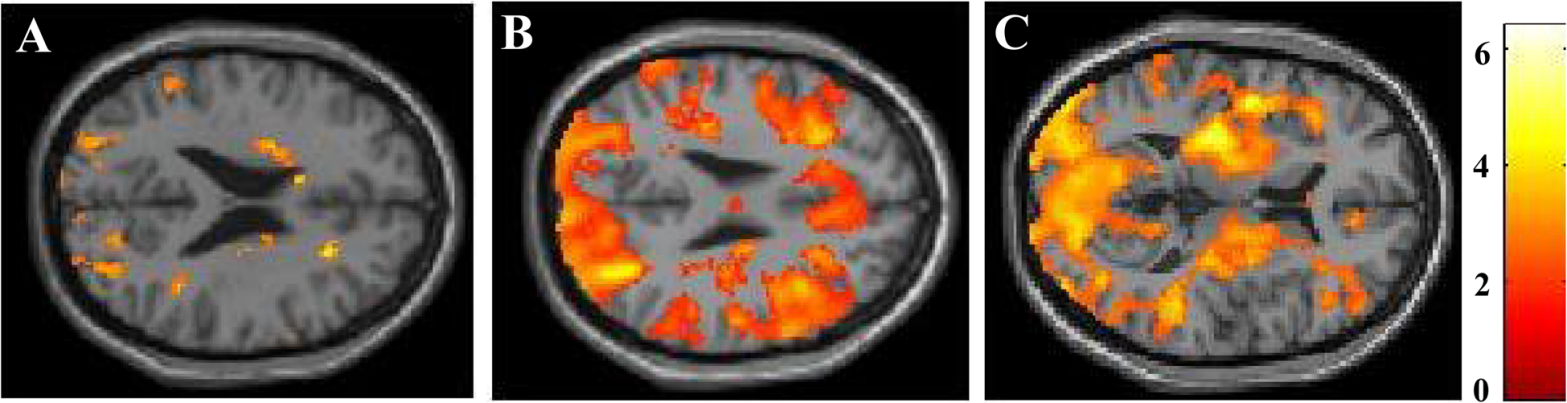
fMRI analyses comparing the patients with PD and HC. The figure shows the results for the 0-back test (**A**), 2-back test (**B**) and subtracting the activated regions in the 0-back test from those in the 2-back test (**C**). The coloured regions indicate significantly lower brain activation in patients with PD, as compared with HC. The colour-bar represents t values as reference. All the images presented at *P* < 0.01 (uncorrected) with cluster size > 50 voxels in analysis. (**A**) The fMRI result during the 0-back test showed only a marginal difference in frontal lobe. (**B**) The result of the 2-back test showed the reduced activation in the bilateral dorsolateral prefrontal cortex, middle frontal gyrus, inferior parietal lobule, and superior parietal lobule. (**C**) The result of 2-back versus 0-back condition presented reduction of the activation in the bilateral prefrontal cortex, occipital cortex, and bilateral putamen

First, fMRI group analyses between the patients with PD and HC revealed significant reduction of the brain activation in the patients with PD, comparing with normal subjects. The images of significantly decreased voxels of the n-back test in the patients with PD are presented in Fig. 3. The result of the 2-back test showed the reduced activation in the bilateral dorsolateral prefrontal cortex, middle frontal gyrus, inferior parietal lobule, and superior parietal lobule. In contrast, the result of the 0-back test showed only a marginal difference in frontal lobe. The result of 2-back versus 0-back condition presented reduction of the activation in the bilateral prefrontal cortex, occipital cortex, and bilateral putamen.

**Fig. 4:**
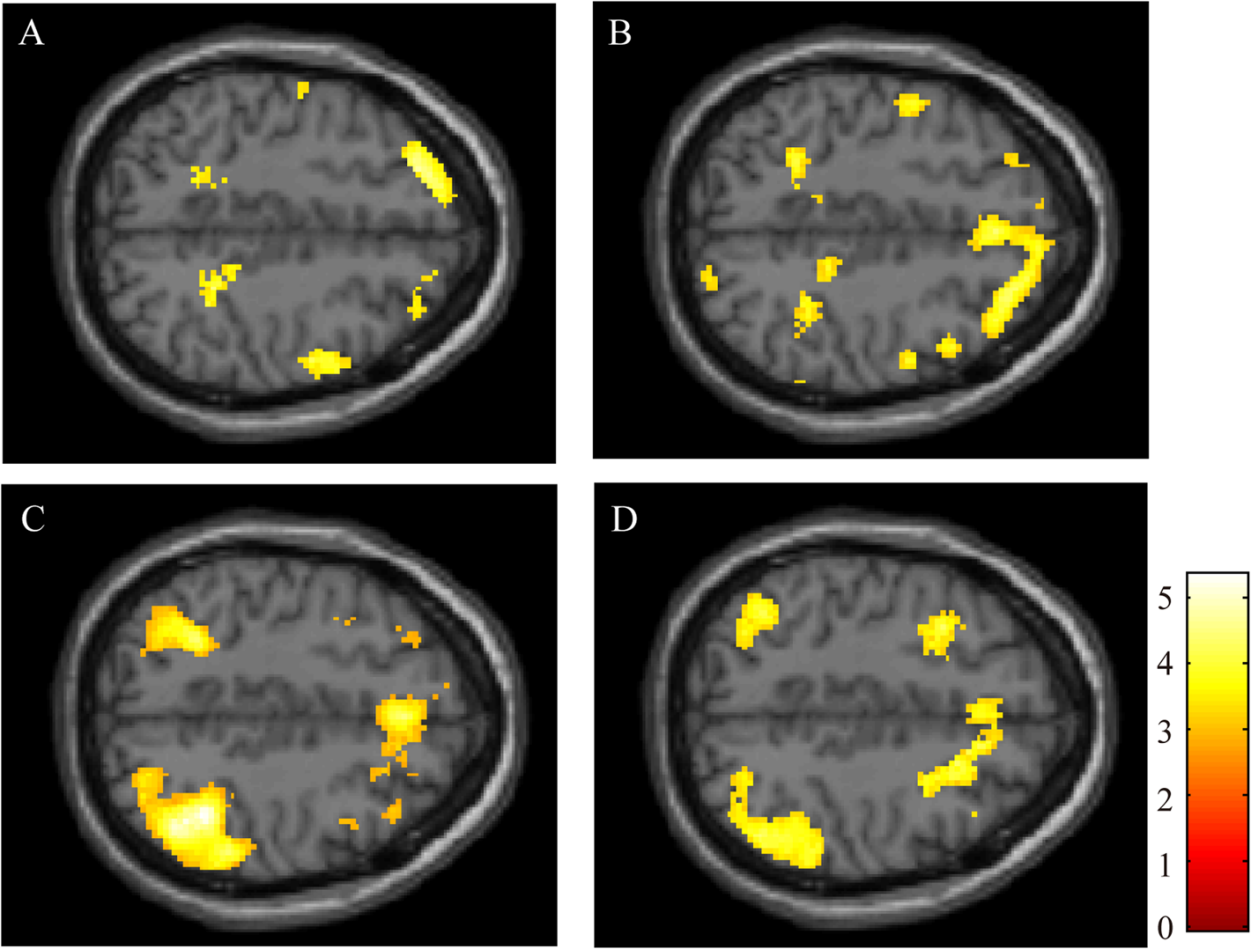
fMRI analyses comparing PD-MCI and PD-CN. These images show the results for the 0-back test (**A**), 1-back test (**B**), 2-back test (**C**), and subtracting the activated regions in the 0-back test from those in the 2-back test (2-back versus 0-back condition) (**D**). The coloured regions indicate significantly lower brain activation in PD-MCI, as compared with PD-CN. The colour-bar represents t values as reference. All the images presented at *P* < 0.01 (uncorrected) with cluster size > 50 voxels in analysis

In next step, we did group analyses to compare the patients with PD-MCI and the patients with PD-CN. In comparisons between PD-MCI and PD-CN, MNI coordinates of the centre of activation and cluster size for significant voxels are summarised in Table 4. For the 0-back test, the PD-MCI group presented significantly reduced brain activation within the right inferior frontal gyrus, left superior frontal gyrus and left medial frontal gyrus. For the 1-back test, significant differences between the groups were observed for right IFG, bilateral superior parietal lobule and bilateral cuneus. For the 2-back test, the PD-MCI group presented significantly reduced brain activation within right inferior parietal lobule (IPL), right middle frontal gyrus (MFG), left superior parietal lobule, left lateral globus pallidus, left cerebellar tonsil and left precentral gyrus. For the 2-back versus 0-back condition, the patients with PD-MCI presented reduced activation within the bilateral MFG and IPL, compared to the patients with PD-CN. These images of significant voxels for each load of the n-back test are presented in Fig. 4.

**Table 4:** MNI coordinates of the centre of activation for each load of the n-back test in comparisons between PD-MCI and PD-CN

Brain region	Side	Cluster size	x	y	z	T-value
**0-back test**						
Inferior Frontal Gyrus	R	395	32	16	−18	3.96
Superior Frontal Gyrus	L	357	−24	40	44	4.13
Medial Frontal Gyrus	L	250	−8	48	10	3.45
**1-back test**						
Inferior Frontal Gyrus	R	847	28	20	−22	3.99
Superior Parietal Lobule	R	820	30	−52	56	3.98
Inferior Semi-Lunar Lobule	L	714	−18	−64	−36	4.14
Cuneus	R	654	14	−82	26	4.11
Middle Occipital Gyrus	R	543	44	−82	16	3.78
Superior Temporal Gyrus	L	487	−44	−40	10	4.30
Cuneus	L	422	−12	−86	28	4.00
Fusiform Gyrus	R	353	42	−44	−16	3.84
Superior Parietal Lobule	L	342	−30	−50	52	3.32
**2-back test**						
Inferior Parietal Lobule	R	2008	40	−48	46	4.72
Middle Frontal Gyrus	R	1602	44	24	36	4.49
Middle Frontal Gyrus	R	753	38	46	−14	4.32
Superior Parietal Lobule	L	752	−28	−50	46	3.91
Lateral Globus Pallidus	L	643	−24	−8	2	3.92
Cerebellar Tonsil	L	457	−30	−62	−38	3.12
Precentral Gyrus	L	436	−62	14	8	4.78
**2-back test–0-back test**						
Inferior Parietal Lobule	R	1031	52	−36	40	3.74
Middle Frontal Gyrus	L	810	−32	22	32	4.27
Middle Frontal Gyrus	R	621	28	16	56	3.85
Middle Frontal Gyrus	R	386	42	22	40	3.80
Inferior Parietal Lobule	L	320	−36	−54	38	3.49
Middle Occipital Gyrus	R	312	26	−72	6	3.91

Coordinates x, y and z refer to the anatomical location of the Montreal Neurological Institute space for local maxima of clusters

**Fig. 5:**
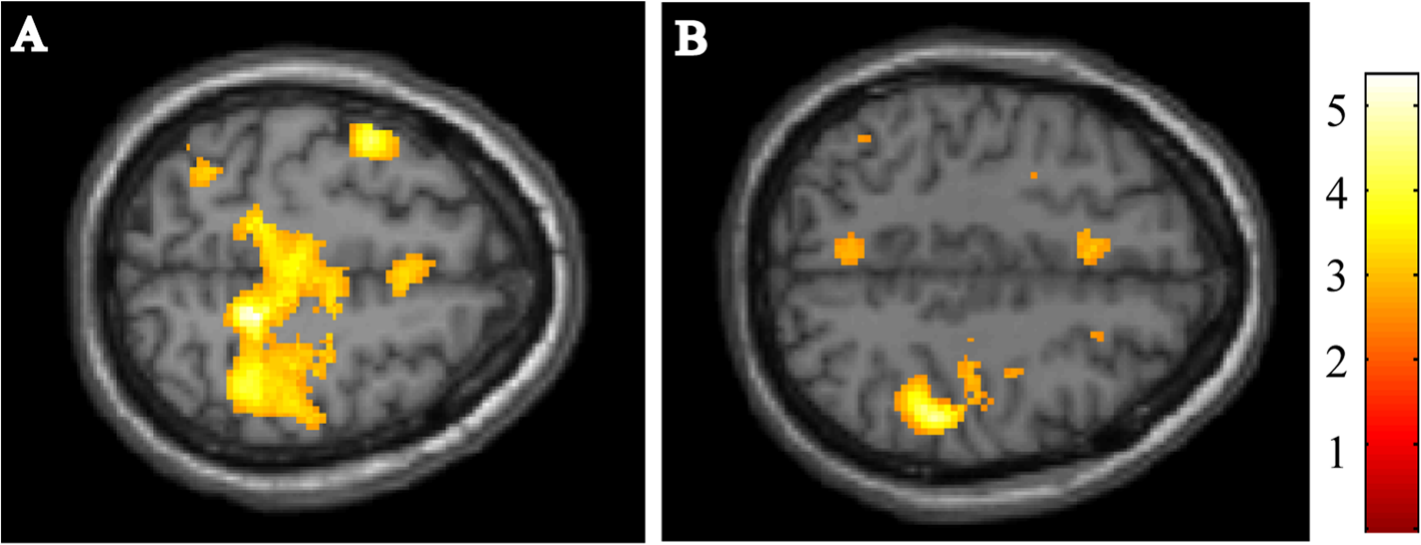
fMRI correlation analyses for the patients with PD-MCI. The figure shows the results of correlations between the scores on the n-back test and task-related activation in the PD-MCI group. The colour-bar represents t values as reference. All the images presented at *P* < 0.001 (uncorrected) with cluster size > 50. **A**. The image shows the correlation between the scores of the 1-back test and activation of the 1-back test. **B**. The image shows the correlation between the scores of the 2-back test and activation of the 2-back test

The correlations between the scores of the n-back test and task-related activation in the PD-MCI group are shown in Fig. 5. We found a positive correlation between the score of the 2-back test within the right IPL and the scores of the 1-back test within superior parietal lobule and MFG.

## Discussion

This study aimed to investigate the visuospatial working memory and fMRI findings of the patients with PD-MCI and PD-CN, using the visuospatial n-back test. The scores of the 0-back and 1-back test did not differ significantly between groups. By contrast, the scores of the 2-back test in the patients with PD-MCI was significantly lower than PD-CN. It suggested more severe impairment of the visuospatial working memory in the patients with PD-MCI. Further, the patients with PD-MCI showed the reduced brain activation within bilateral MFG and IPL which was associated with impairment of visuospatial working memory.

Concerning PD-MCI, a meta-analysis reported the prevalence of PD-MCI as 26 % [23], and another reported as 55 % among patients with mean disease duration > 10 years [24]. In further, approximately 10 % of patients with PD convert from MCI to dementia each year [25]. Therefore, the diagnosis of PD-MCI and cognitive assessment including working memory are important for clinical management. The cognitive impairment in PD is caused by complex pathology. Diffuse Lewy bodies [26–28], Alzheimer disease [27, 28], loss of cholinergic neurones [29], loss of medial nigral dopaminergic neurones [30], and serotonergic and noradrenergic deficits [31] are implicated in dementia in PD.

Recently, various neuroimaging studies are focusing on PD-MCI to examine which type of pathology is a dominant cause of cognitive impairment. Some studies using quantitative susceptibility mapping have indicated that brain iron deposition is linked with cognitive severity in PD [32, 33]. Thomas et al. reported that whole brain regression analyses of PD identified QSM increases that covaried with lower MoCA scores in the hippocampus and thalamus, with poorer visual function, and with higher dementia risk scores in the parietal, frontal, and medial occipital cortices [33]. To search the brain regions associated with cognitive function in patients with PD, the MoCA has mostly been used as a covariate in recent neuroimaging research. However, the MoCA may be unsuitable as a covariate of neuroimaging studies that explore the brain areas involved in executive function, because it is not a test for single cognitive function; it is a global cognitive test consisting of five different cognitive subdomains. In contrast, the visuospatial n-back test is advantageous as patient verbal ability has a smaller impact on the test score, in comparison to other cognitive tests, including the MoCA. For this reason, we selected the visuospatial n-back test as an activation task of fMRI to identify the brain regions which associated with working memory in the patients with PD-MCI and PD-CN.

In the present study, fMRI analysis of patients with PD and HC during the 2-back test revealed that patients with PD showed reduced functional activations in the bilateral dorsolateral prefrontal cortex, middle frontal gyrus, inferior parietal lobule, and superior parietal lobule. However, the results of the 0-back test identified no specific region that was activated. The plausible reason for this difference may be dependent on working memory demand, i.e., the 2-back test requires sustained attention and visuospatial working memory at a high level, while the 0-back test requires sustained attention but not working memory [34]. The results of the correct answer rates in the n-back tests revealed that patients with PD had inferior performance than HC in all task conditions. However, compared with PD-CN, PD-MCI showed significantly poorer performance only in the 2-back test, which required visuospatial working memory at a high level. Focusing on fMRI analysis comparing the PD-MCI and PD-CN groups, the result of the 2-back test indicated significantly reduced brain activation within the right inferior parietal lobule (IPL) and right middle frontal gyrus (MFG) in patients with PD-MCI. However, these areas were not identified as being activated based on the results of the 0-back and 1-back tests. Considering these results, deactivation within the IPL and MFG represents impaired visuospatial working memory in patients with PD-MCI. Furthermore, subtracting the activated regions in the 0-back test (the easiest condition) from those in the 2-back test is adequate for identifying specific brain areas associated with working memory demand. However, the subtracting condition of the 0-back test from those of the 1-back test had limited statistical power to detect brain areas associated with working memory because the 1-back test requires low-grade visuospatial working memory. A similar interpretation of the fMRI analysis has also been reported in a previous study on visuospatial working memory in patients with attention-deficit/hyperactivity disorder; the increase in task-related activation was the most prominent for the 2-back condition, while the difference between the 0-back and 1-back tests was limited [35].

Herein, we discuss about the deactivation of the MFG and IPL. First, concerning the IPL, one post-mortem autopsy report demonstrated that patients with PDD had reduced levels of dopaminergic transporter in the caudate, precuneus, and IPL [20]. Another study that used connectivity analysis of resting-state fMRI to target newly diagnosed PD revealed that patients with PD-MCI showed decreased functional connectivity between the posterior cingulate cortex and posterior IPL [36]. Second, concerning the MFG, Xu et al. reported that the fronto-striatal functional connectivity degree in the right globus pallidum was negatively correlated with that in the left MFG and disease duration [37]. Based on these reports, reduced activation within MFG and IPL in the present study may have been caused by disease-associated dysfunction of the cortico-striatal neural network in PD.

Various studies report conflicting results of the fMRI during tasks that require working memory. A recent fMRI study that incorporated the n-back task reported that functional activity of de novo patients with PD, compared with controls, was increased in right dorso-lateral prefrontal cortex (including MFG) [38]. The results suggest compensation to maintain behavioural performance in the presence of de novo network deficits. In contrast, Alex et al. reported that the patients with PD have reduced neural interactions between left prefrontal executive circuits and the left supramarginal/superior temporal cortices during the stimulus encoding phase which may underlie their diminished working memory [39]. Furthermore, Simioni et al. reported that patients with PD who were off dopamine replacement therapy displayed reduced activation in prefrontal and bilateral parietal cortex, and revealed that L-DOPA seems to both boost engagement of a task-specific prefrontal region and strengthen a putative compensatory caudate-cortical network of the patients with PD [40]. These reports may suggest that the deactivation of MFG and IPL found in our study are associate with the pathophysiology of working memory impairment in PD which depend on the network dysfunction.

This study had some limitations. First, performance of neuropsychological tests for executive function is influenced by several factors. Disease-associated impairment of working memory, as well as psychosis, depression and daytime sleepiness, may affect performance. The visuospatial n-back test, in particular, requires continuous attention. Increased variability in subjects’ performance on the 2-back test, compared with the 1-back test, results in decreased statistical power. Second, there is a possibility that the neuroimaging results of n-back tests were also affected by emotions, such as anxiety and depression, because emotions influence the modulation of the selectivity of attention as well as the motivation of action and behavior. To reduce the influence of depressive mood, we excluded participants who had depression at the time of registration. In addition, we performed fMRI scanning of the patients with PD during the ‘On’ state to avoid cognitive change or anxiety arising from being in the ‘Off’ state. Although there is no specific way to quantify real emotions during the task performed inside the MRI scanner, we think these methods contributed to reducing the influence of negative emotions on the neuroimaging results. Third, although the L-DOPA and the LEDD doses were similar between groups, we cannot rule out the possibility that variety of the dopaminergic agonist may have had differing impacts on performance. Fourth, because the present study had a relatively small sample size, the statistical power to analyze the neuropsychological data of the n-back test and to search for significant voxels in the fMRI analysis was limited. For imaging analysis, we used a 10 mm smoothing kernel in analysis. Although there is no easy answer regarding the extent to which we should smooth the imaging data, the main disadvantage of using higher smoothing kernel is the loss of spatial specificity. However, because of the small sample size, we had to reduce noise as much as possible; therefore, we used a relatively high smoothing kernel compared with the voxel dimension. For this reason, we used a 10 mm smoothing kernel instead of the reduction of spatial resolution, and we could not find significant voxels in the imaging analysis with family-wise error correction.

However, this study has two strengths. First, the stringent quality control of clinical and neuropsychological assessment has done in this study. Second, the visuospatial n-back test can assess both visuospatial recognition and working memory in a single test with three different loads. This fMRI protocol can be used to explore the pharmacological mechanisms in the central nervous system noninvasively. In further study using this protocol, the effect of anti-dementia medication may be demonstrated by the changes of brain activation comparing between anti-dementia medication and placebo.

## Conclusions

We revealed the reduced activations within MFG and IPL which was associated with the impaired visuospatial working memory in the patients with PD-MCI. Combinations of functional neuroimaging and the visuospatial n-back test are beneficial to evaluate the impairment of working memory in the patients with PD.

## Data Availability

The datasets used in this study are available from the corresponding author on reasonable request.

## Abbreviations

MDS: Movement disorder society
fMRI: Functional magnetic resonance imaging
PD: Parkinson disease
PD-CN: PD with cognitive normal
PD-MCI: PD with mild cognitive impairment
MoCA: Montreal Cognitive Assessment
TMT-A: Trail-Making Test Part A
TMT-B: Trail-Making Test Part B
PASAT: Paced Auditory Serial Addition Test
AVLT: Auditory Verbal Learning Test
IPL: Inferior parietal lobule
MFG: Middle frontal gyrus
